# 

**Published:** 1894-06

**Authors:** 


### CONTENTS.

ORIGINAL COMMUNICATIONS—

Appendicitis, with Report of Cases. By Floyd W. McRae, M.D., Atlanta, Ga... 193
Making and Closing the Incision in Abdominal Surgery. By J. B. S. Holmes, M.D., Atlanta, Ga........ 199
Malignant Growths of the Skin: Their Diagnosis and Treatment. By M. B. Hutchins, M.D., Atlanta, Ga........ 205
Orator's Address before the Alabama Medical Association, April 17th, 1894. By W. H. Blake, M.D., Lineville, Ala........ 212

SOCIETY REPORTS—

Atlanta Society of Medicine........ 223

CORRESPONDENCE—

Our New York Letter........ 234

EDITORIAL—

Some Homeopathic Therapeutics........ 239
The Amick Quack Company........ 240
Foot-Ball Casualties........ 242

SELECTIONS AND ABSTRACTS—

Practical Chemistry and Pharmacy........ 244
Skin Diseases and Syphilis........ 247

MEDICAL ITEMS........ 250
BOOK REVIEWS........ 253
ADVERTISERS' INDEX TO ADVERTISEMENTS........ xiv
CLASSIFIED INDEX TO ADVERTISEMENTS........ xxxvii
ITEMS OF INTEREST........ x, xi, xii, xxviii, xxix

